# Pursuing decarbonization along with national security: Assessing public support for the Thacker Pass lithium mine

**DOI:** 10.1371/journal.pone.0280720

**Published:** 2023-01-24

**Authors:** Azusa Uji, Jaehyun Song, Nives Dolšak, Aseem Prakash

**Affiliations:** 1 Graduate School of Law, Kyoto University, Kyoto, Japan; 2 Faculty of Informatics, Kansai University, Osaka, Japan; 3 School of Marine and Environmental Affairs, University of Washington, Seattle, Washington, United States of America; 4 Department of Political Science, University of Washington, Seattle, Washington, United States of America; Central Queensland University, AUSTRALIA

## Abstract

Decarbonization policies are being stymied by political conflict. Local communities might oppose decarbonization infrastructure such as solar farms, mines, or transmission lines if they view these projects as imposing high costs on them in relation to their benefits. To decarbonize, the automobile industry seeks to shift from the internal combustion engine to electric vehicles, which require lithium-based rechargeable batteries. In the United States, to meet the increasing lithium demand through domestic sources, there is a proposal for a lithium mine in Thacker Pass, Nevada, which faces strong opposition from native nations and environmental groups. Using a representative sample of Nevada residents (n = 1,368), we explore if proximity to the Thacker Pass mine and to any Nevada mine influence public support for the proposed lithium mine. In addition, we test three frames that emphasize different benefits of the proposed mine: climate policy, national security, and local economic development. We find that respondents living closer to the Thacker Pass mine tend to be more supportive of the proposed lithium mine but exposure to existing Nevada mines does not affect public support. Among the treatment frames, only the national security frame increases public support. This suggests that to navigate local public opposition, the national security—domestic sourcing of key inputs required for decarbonization, aspect of decarbonization projects should be highlighted.

## Introduction

The transportation sector accounts for 30% of CO_2_ emissions worldwide (Barringer 2021). To decarbonize it, governments and automobile companies have outlined aggressive plans to transition from internal combustion engine (ICEs) cars to electric vehicles (EVs). Although some scholars are concerned about the high carbon intensity of EV components and manufacturing, and EVs’ carbon footprint from the cradle to the grave [1, 2], several countries have announced bans on the sale of new ICEs: Norway by 2025, Denmark, Ireland, and Israel by 2030, France and China by 2040, and Germany by 2050 [3]. Even in the U.S., typically a laggard in climate policy, some states have announced ICE bans: Washington by 2030 and California by 2035.

Some project that 31 million EVs (and plug-ins) will be sold worldwide by 2030. To support this massive volume, policymakers seek to outline their “lithium strategy.” The reason is that EVs are powered by batteries, of which lithium is a key component. The International Energy Agency projects that lithium demand will grow at least 13-fold by 2040 [4]. Indeed, the price of lithium has increased from about $10,000 metric tons in December 2020 to about $70,000 metric tons in December 2022 [5]. While some companies have developed technologies to recycle lithium [6, 7], currently only 2–5% of lithium-ion batteries are collected in Australia, the EU, and the US due to the lack of consumer awareness and legal and physical infrastructure [8]. Because existing mines and recycling infrastructure cannot meet the increased demand, new mines will need to be commissioned. While the US has abundant domestic lithium reserves, it has only one large-scale lithium mine, Silver Peak in Nevada, which opened in the 1960s. In recent years, there are proposals for new lithium mines in Nevada, California, Oregon, Tennessee, Arkansas, and North Carolina [9].

Decarbonization processes are being stymied by political issues, namely the perceptions about the asymmetrical distribution of costs and benefits across actors [10–12]. Local communities might oppose decarbonization infrastructure such as solar farms, mines, or transmission lines if they view these projects as imposing high costs on them in relation to their benefits. Despite the climate imperative, opening new mines could encounter opposition from local communities who often view new mines to be causing environmental pollution and water shortages. Along with health issues, new mines could decrease property values. This anti-mining local sentiment is visible in the context of proposed lithium mines in the Thacker Pass in Nevada and the Big Sandy River Valley in Arizona [4].

On the other hand, local communities might welcome new mines. After all, mining is central to the livelihood and culture of many communities. Local people may regard new lithium mines as creating economic benefits and supporting the local economy. The remark of the County Commissioner of Gaston County in North Carolina, where a new lithium mine is proposed, reflects the complex nature of benefits and costs: “If it’s just about money, [the mine] is the greatest thing, because it brings jobs and brings money and brings glorious things…But it’s not just that, it has to do with the environment, the quality of life for the people who are surrounding it” [13].

The tension between decarbonization and local opposition is not limited to lithium. Rare earth elements like nickel, cobalt, and manganese are also essential to EVs and renewable energy technologies. China controls 70% of global supplies of rare earth elements and the US is fully dependent on imports of the elements. Like lithium, mining these minerals has negative impacts on local land, water, and wildlife. Because gaining local support for mining is critical for decarbonization, it is important to understand the political and economic dynamics behind both the local support and opposition to new mines.

Our study on local support/opposition to asymmetric decarbonization costs of opening a lithium mine speaks to the literature on not-in-my-backyard (NIMBY). Studies suggest that communities living in proximity to projects with negative local externalities such as electricity generation facilities, transmission lines, and waste facilities, oppose them [14, 15]. The idea is that while individuals might appreciate the public good benefits of these projects, they nevertheless oppose their siting in their backyards, given their negative local externalities. However, the literature also notes that sometimes these projects draw local support (yes-in-my-backyard or YIMBY) because local communities view their potential benefits as exceeding costs [16, 17]. Arguably, the local response is also shaped by the framing of the benefits and costs of such projects.

While there is a rich NIMBY/YIMBY literature examining energy projects and waste facilities, NIMBY/YIMBY issues in mining projects are relatively less explored. In exploring state-level support for a lithium mine, we consider where people live in relation to the proposed mine. Because prior exposure to some other mining activity might shape their support for the proposed Thacker Pass mine, we take into account their proximity to any mine in Nevada. Further, we investigate how the focus on different types of benefits (climate, national security, and economic development) shapes public support for the proposed mine.

Nevada has a sizeable mining industry [18]. While Nevada is best known for its world-class deposits of gold, silver, and copper production, it is also a significant source of minerals, such as lithium, iron, and molybdenum. Nevada has abundant industrial minerals such as gypsum, limestone, sand, and gravel. Hence, the mining industry has been vital to Nevada’s economy. More than 12,000 people are directly employed by the Nevada mining industry, mainly in rural Nevada, and earn the highest annual salaries in the state, $83,000 on average. In addition, the economic spillover of mining is remarkable: approximately four other jobs provide goods and services for every mining job. Notably, the mining industry, which is a vital source of technological advancement, has been driving innovation in engineering and science in Nevada [19].

The Thacker Pass lithium mine, located in Nevada’s Humboldt County, is one of the largest known lithium deposits in the world [20]. Some suggest that it could even meet 25% of global lithium demand. While the state-level permits were issued in February 2022, the project faces a federal lawsuit filed by a coalition of indigenous communities, environmental groups, and ranchers. In particular, the opposition from indigenous groups is salient. Northern Nevada, where the Thacker Pass lithium mine project is located, was inhabited by the Northern Paiute for as long as 15,000 years. Several native nations have asked the U.S. courts for intervention to halt the project arguing that ancestral graves are in the zone of the construction and thus Thacker Pass is a sacred site. As one of the protesters camped outside the proposed mine said, “Environmentalists might be confused about why we want to interfere with the production of electric car batteries… But, it’s wrong to destroy a mountain for any reason—whether the reason is fossil fuels or lithium.” Another activist notes: “To protect what’s left of the natural world, we must leave minerals in the ground—not just oil and gas—and learn to live within ecological constraints as respectful members of natural communities. Humans must learn to live with less. Or, we will not live at all” [21].

The suit alleges that the Bureau of Land Management conducted a flawed environmental review. This is a serious issue because the project is expected to extensively use and contaminate precious groundwater. It will also generate mining waste that may contain radioactive uranium. Finally, the mine could harm the habitat of threatened species such as the Greater Sage Grouse, Lahontan cutthroat trout, and Pronghorn antelope [9]. Moreover, the suit alleges that there was inadequate consultation with tribes [22], although the proposed mine will despoil lands fundamental to their history and culture [23]. Native groups contend that BLM violated several federal laws including the National Historic Preservation Act which requires the BLM to identify historic sites. Thacker Pass is the site of two massacres involving Native people and the proposed mine would disturb a cemetery.

To assess local public support for the proposed Thacker Pass mine, we administered a survey-embedded experiment to a representative sample of Nevada residents (n = 1,368). We divided respondents into four groups (a reference group and three treatment groups) and randomly assigned them to different treatment frames focusing on specific benefits of the Thacker Pass lithium mine (climate policy: global public good, national security: national public good, and local economic development: local public good). Overall, we find that respondents living closer to Thacker Pass tend to be more supportive of the mine (YIMBY). Among the treatment frames, only the national security frame increases public support (the coefficient of the economic development frame is positive but of the climate frame is negative; neither is statistically significant). This suggests respondents are willing to incur costs to provide for a national-level public good when it ‘butes to national security, which is threatened by China’s domination of the lithium market. Much to our surprise, exposure to Nevada’s 165 mines does not affect public support for Thacker Pass.

Our findings suggest policymakers should analyze local opposition to new mines that are critical to decarbonization in terms of NIMBY/YIMBY dynamics. Further, the national security frame can provide a new avenue to highlight a new dimension of decarbonization that could mitigate local opposition. This paper is organized into five sections. In section two, we review the literature on NIMBY/YIMBY and mining and propose several hypotheses. In section three, we discuss our data, survey experiment, and statistical model. We present the results in section four and conclude with a discussion of the paper’s contributions and limitations in section five.

## Theory and hypotheses: Mining, NIMBY, and YIMBY

Does support for new projects that impose local costs—environmental, health, aesthetic, or economic—but generate public goods benefits (that spill beyond the local jurisdiction) depend on where you live? Scholars suggest that such projects provoke NIMBY opposition from local communities. They have documented this opposition to wind projects [14–16, 24, 25], solar projects [29], and nuclear energy [26–32]. Others report this opposition concerning prisons [33], nuclear waste repositories [34, 35], and waste facilities [36–38]. However, other scholars have noted instances of “YIMBY,” in which local communities support “undesirable” industries in their backyards, primarily to secure local economic benefits [16, 17, 34, 39–43].

There is a literature examining NIMBY in the mining context [44–50]. Only Martinez-Alier (2001) partially discusses the US case among them, and none of these explore public attitudes by surveys [49]. There are two exceptions that assessed public support for mining in relation to NIMBY/YIMBY outside the US. Van der Plank et al. (2016) investigated local attitudes toward a proposed sand mine in rural Victoria, Australia [51]. Pelekasi et al. (2012) assessed the local community’s willingness to accept compensation for marble quarries in Greece [52].

Our study is distinct from these mining-focused studies in four ways. First, theoretically, we explore support for the Thacker Pass mine in the context of two major international developments: climate change and China’s emergence as a major global power and the dominant supplier of critical minerals. Second, while these studies target a limited number of residents living in the proximity of a proposed mine, our study targets a large sample of residents in the whole of Nevada state (N = 1368), with varying distances from the mine (as opposed to imposing arbitrary cutoffs regarding the definition of local). This is important because the proposed Thacker Pass lithium mine has an enormous scale with potentially substantial economic and political impact across the state.

Third, we introduce a novel measure of exposure (or proximity) to a large number of existing mines, which might sensitize individuals to the benefits and costs of mining. Nevada with over 165 working mines, provides an excellent opportunity to assess the “exposure leads to opposition or support” thesis. Indeed, we can test how proximity to Thacker Pass *alongside* exposure to existing working mines might influence public support for the Lithium mine. Fourth, unlike other studies, our paper employs a survey experiment, which allows us to explore the level of public support as respondents learn about different types of benefits from the Thacker Pass mine. These benefits pertain to different scales at which the mine will create public goods: global (climate change), national (national security), and local (economic development).

### Hypotheses

As discussed above, the conventional NIMBY literature suggests that communities living in the vicinity of a proposed facility oppose it because they perceive it as imposing local costs while generating benefits for non-local communities. YIMBY, in contrast, suggests that while the facility imposes local costs, it also creates local benefits of a higher magnitude. Consequently, those living in the vicinity of the facility support it. In either case, the level of support will be associated with proximity to the facility.

Based on the above discussion, we propose:

H1a: Support for the Thacker Pass mine is lower among respondents living in its proximity (NIMBY).
H1b: Support for the Thacker Pass mine is higher among respondents living in its proximity (YIMBY).

Might NIMBY or YIMBY depend on whether individuals have first-hand knowledge of the harms or benefits of mining? Some scholars argue that a crucial driver of energy policy preferences is first-hand exposure to energy facilities located in physical proximity [53]. This insight is especially important in assessing support for the Thacker Pass project because the state of Nevada has 165 working mines. Thus, we expect a large number of Nevada residents probably have some sort of first-hand experience of how mining activities might affect local communities. We, therefore, propose:

H2a: Support for the Thacker Pass mine will be associated with proximity to working mines.
H2b: Opposition to the Thacker Pass mine will be associated with proximity to working mines.

Might the support for Thacker Pass change if a specific type of benefit is highlighted? The literature suggests that non-NIMBY factors such as the even non-local benefits and costs of energy projects influence local public support [54–56]. First, under the Paris Agreement, the U.S. has pledged to reduce greenhouse emissions by 50–52 percent by 2030 (with 2005 as the baseline) [57]. To achieve net-zero emissions no later than 2050, the U.S. transportation sector (predominantly automobiles) will need to be decarbonized [58]. These sorts of aggressive decarbonization targets have been adopted by a large number of countries.

This means that opening new lithium mines is crucial to achieving this massive transformation of the automobile sector. Because opinion polls suggest that a majority of Americans support climate action [59, 60], might survey respondents see the Thacker Pass mine helping the climate cause? On the other hand, climate mitigation creates a global public good that benefits everyone, including individuals living outside Nevada, while the costs of Thacker Pass mining are borne by local communities. Given the mismatch between the geographical focus of benefits and costs, Nevada residents might be less persuaded by the global public goods type of climate benefits of the Thacker Pass mine.

Second, scholars note that climate change has important implications for U.S. national security. Since 2010, the Pentagon has issued several reports on this subject. In recent years, another aspect of national security has received policy and media attention: dependence on foreign suppliers for critical inputs for the domestic economy. This national security dimension received a lot of attention during the COVID-19 epidemic and more recently, the Ukraine invasion. As global supply chains were disrupted, the US economy suffered, which threatened national security. Because the US is dependent on foreign suppliers for lithium, national security issues have become implicated in the Thacker Pass debate. Most of the lithium is mined in Latin America or Australia and processed and turned into battery cells in China and other Asian countries [9].

The heavy reliance on China and other foreign countries for critical minerals like lithium is increasingly regarded as a matter of national security. Even the Trump administration, which was hostile to climate policy, took steps to reduce U.S. dependence on foreign suppliers for critical minerals. Recently the Biden Administration invoked the Defense Production Act, which authorizes the federal government to provide financial support for projects such as the mining and processing of minerals required for EV batteries [61]. Yet, national security constitutes a national-level public good, and Nevada residents might not want to bear local costs to reduce U.S. dependence on foreign lithium suppliers.

Third, mines create economic benefits for local communities [62]. New mines attract a large volume of investments and create jobs both in the construction phase and eventually in mining and processing facilities. Population growth supports the retail sector. Mines also create tax revenue for local governments, which helps fund schools, roads, and public services [4].

In sum, we expect that exposing respondents to different types of benefits from the Thacker Pass mine can enhance their support/opposition.

H3a: Support for the Thacker Pass mine will increase when respondents are provided information that the new mine is essential for *climate policy*.
H3b: Support for the Thacker Pass mine will increase when respondents are provided information that the new mine is essential for *US national security*.
H3c: Support for the Thacker Pass mine will increase when respondents are provided information that the new mine contributes to *local economic development*.

## Data and methods

We administered an online survey to a representative sample of Nevada citizens who are 18 or older. We received IRB approval from the University of Washington’s Human Subjects Division (#STUDY 00014935), and we pre-registered our survey (https://osf.io/kghtf).

We contracted with a survey firm, Lucid, to administer the online survey between February 24–March 1, 2022. At the start of our survey, we obtained written informed consent from survey participants. As shown in Table A1 in S1 Appendix, our sample is fairly representative of Nevada’s population of the 2020 American Community Survey (ACS) in terms of age and regional salience (by county). However, our sample is less representative in terms of gender. We, therefore, weighted the data so that it corresponds to the gender distribution reported in the 2020 ACS census [63]. We use the weighted data throughout our analysis, both in the main text and in the Appendix.

Our survey is structured as follows (the full text is available in Text A4 in S1 Appendix). We randomly assigned respondents to four groups (the reference frame and three treatment frames). Participants first read common information and the general discussion about the increasing demand for lithium for EVs in the context of decarbonization. After the general discussion, we inserted treatment information that highlighted different types of benefits of the Thacker Pass mine. The climate policy frame notes that the United States, the largest contributor to accumulated greenhouse gas emissions, should facilitate lithium mining since it has sizeable lithium deposits. The national security frame highlights that China is the leader in the EV sector, including lithium batteries. To remain globally competitive in the automobile sector and to reduce reliance on China for national security, the United States should support new domestic lithium mines. The local economic development frame notes that the lithium mine will attract a large volume of investments and create well-paying local jobs. This will help the local economy and increase tax revenue for local governments. (See Text A4 in S1 Appendix for full texts of these frames).

After reading these frames, respondents were provided with 2 attention-check questions. After the attention check, we asked respondents to indicate their support for opening the Thacker Pass lithium mine on a 1–7 scale, our dependent variable. Specifically, we asked them to move the slider between 1 (Strongly oppose) and 7 (Strongly support) to indicate their level of support.

Climate politics is embedded in the broader debate on whether environmental protection hurts or supports economic growth. We thus asked how respondents prioritize environmental protection in relation to economic growth. Further, we asked standard demographic questions on gender, age, income, education, race, religion, and political ideology. Finally, we asked for respondents’ residential zip codes to assess proximity to Thacker Pass and proximity to existing mines.

While we administered the survey to 1,697 respondents, 232 (14%) selected the option, “Don’t want to answer” for at least one question. In addition, we dropped 97 inattentive respondents, who were not able to provide correct answers to the two attention check questions. Consequently, the usable sample for the statistical analysis is 1,368. We recognize the debate on the potential bias by excluding inattentive respondents. As reported in Table A2 in S1 Appendix, our substantive findings do not change when we include both attentive and inattentive respondents.

To test our hypotheses, we constructed the following model. Treatment_1_, Treatment_2_, and Treatment_3_ respectively represent each treatment variable. Based on the Nevada Division of Minerals’ mining information portal, we have geocoded 165 existing working mines (for ores, gemstones, and industrial minerals) and oil fields in Nevada state [20]. Combined with the respondents’ ZIP code information, we calculated the respondents’ proximity to the Thacker Pass mine as well as to all existing mines. *Distance_TP* represents the distance (in miles) of the respondent’s county to Thacker Pass. To assess exposure to existing mines, we decided against using an arbitrary cutoff such as the number of mines in the zip code or within say 3 or 5 miles of the zip code. Instead, we decided to calculate the exposure of respondents to all Nevada mines. We calculated *Exposure* as the inverse of the respondent’s distance to all Nevada mines. To illustrate with an example, suppose there are three mines—M1, M2, and M3—in Nevada. Individual A is at a distance of 2, 5, and 40 miles from M1, M2, and M3 respectively. We calculate *Exposure* as the sum of the reciprocals of distances: $0.725=\frac{1}{2}+\frac{1}{5}+\frac{1}{40}$. The variable ranges between 0.764 and 5.271. As a specification check, we also measure the level of exposure to existing mines by the total number of mines in the respondent’s county (*Exposure_County*). The result is available in Table A3 in S1 Appendix. Our main results of the framing experiment hold but the variable *Distance_TP* loses statistical significance.

We also controlled for respondents’ position on the economy-environment debate (*Environment)* and the duration that they had lived in Nevada *(Duration*). This is because we speculate that pro-environment respondents are more likely to support opening the mine, and those who have lived longer in Nevada may be more likely to support (or oppose) opening the mine, acknowledging the benefits (or costs) of having a mine. In addition, we control for demographic variables: *Gender*, *Age*, *Income*, *Education*, *and Party Identification*, which could influence the level of public support for opening the mine. Since using a slider allows the respondent to express their support for the Thacker Pass mine on a continuous scale (bounded between 1 and 7), we employ the OLS estimator.


$$\begin{array}{l} \hat{Y_{i}}=\alpha +\gamma_{1}Treatment_{1,i}+\gamma_{2}Treatment_{2,i}+\gamma_{3}Treatment_{3,i}+\gamma_{4}Distance\_TP_{i}\\ \;+\gamma_{5}Exposure_{i}+\gamma_{6}Environment_{i}+\gamma_{7}Income_{i}+\gamma_{8}Gender_{i}+\gamma_{9}Age_{i}\\ \;+\gamma_{10}Education_{i}+\gamma_{11}Income_{i}+\gamma_{12}Duration_{i}+\gamma_{13}Party\;Identification_{i}, \end{array}$$

## Results

**Fig 1:**
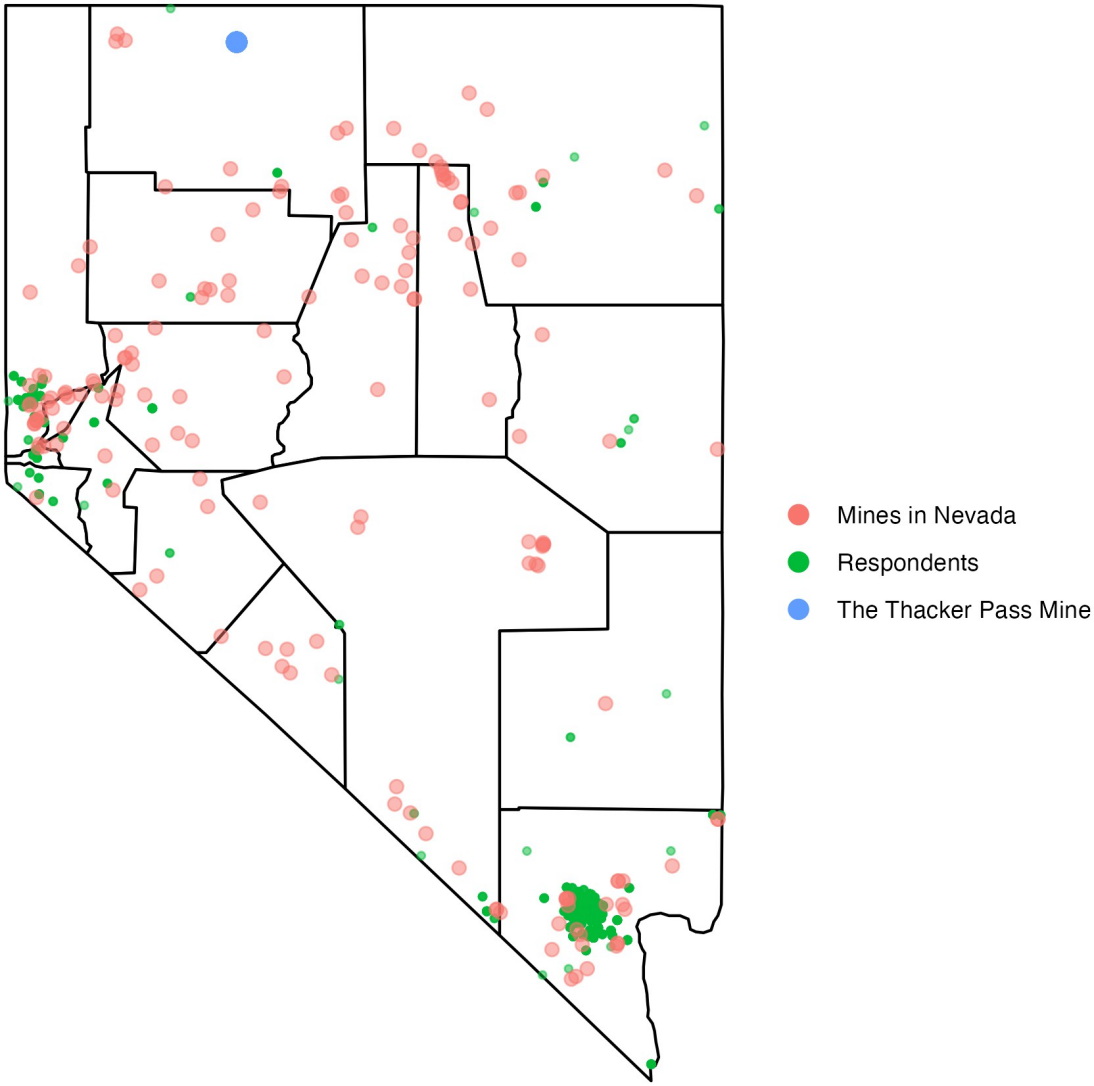
The location of the proposed Thacker Pass lithium mine, existing mines, and respondents.

**Fig 2:**
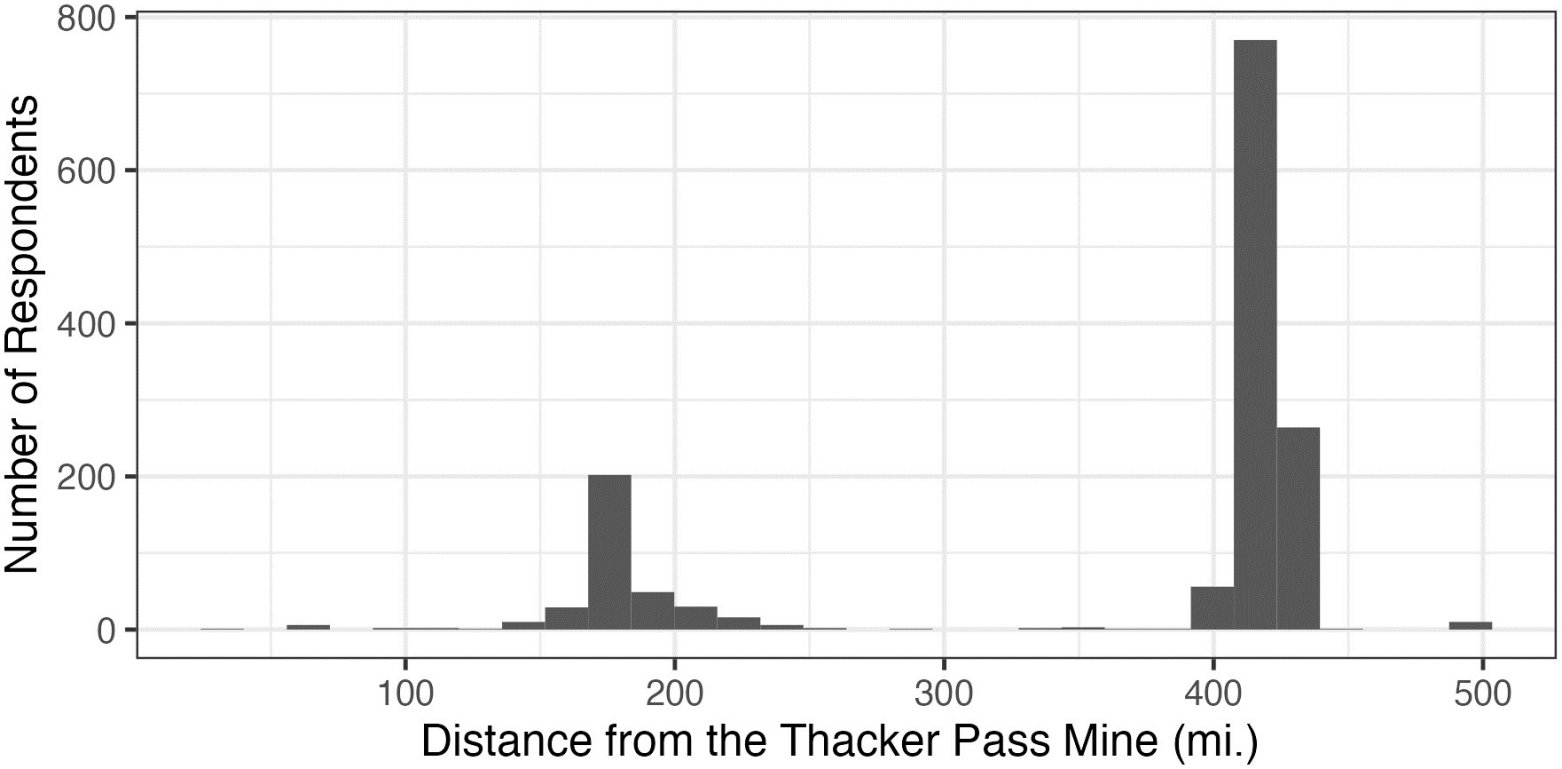
Geographical distribution of respondents in relation to the Thacker Pass lithium mine.

**Fig 3:**
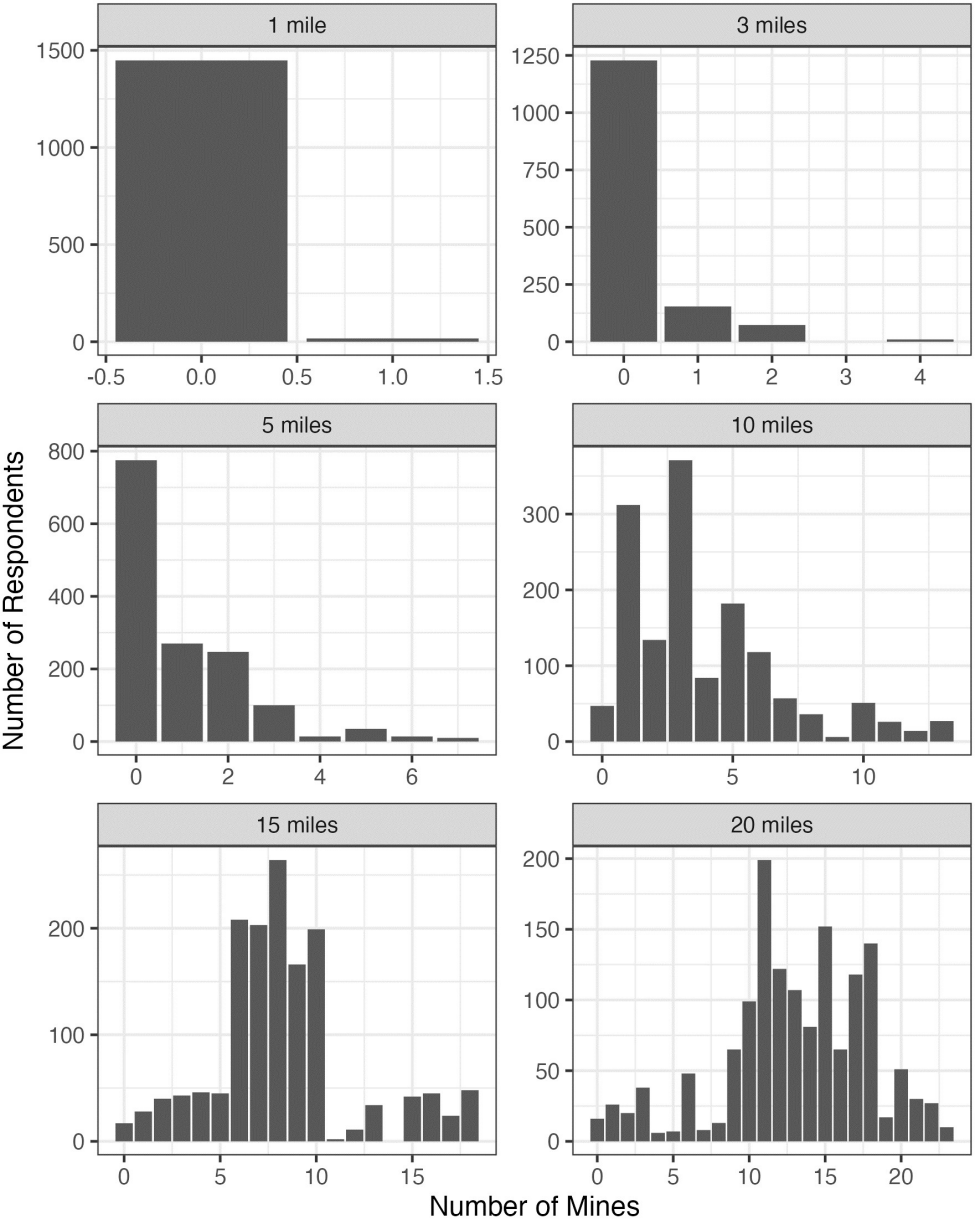
Geographical distribution of respondents in relation to existing mines.

The location of the proposed Thacker Pass mine (blue dots), 165 existing Nevada mines (red dots), and the location of survey respondents (green dots) are mapped (Fig 1). The bars show the number of respondents who reside within a specific distance from the Thacker Pass lithium mine (Fig 2). About 30% of respondents live within 250 miles of the Thacker Pass mine and almost all respondents within 450 miles. Regarding the proximity to existing mines, bars indicate the number of mines that respondents have within 1, 3, 5, 10, 15, and 20 miles of their residence (Fig 3). Panels on the top show that most respondents do not have any mines within 1 and 3 miles. The left-hand side panel in the middle shows that more than half of respondents have at least one existing mine within 5 miles. The right-hand side panel in the middle and panels at the bottom show that most respondents have 1–5, 1–10, 1–18 existing mines within 10, 15, and 20 miles respectively.

Table 1 present the result of our regression model. The coefficient of *Distance_TP* is negative, with a significance level of 0.1. This means that respondents living closer to the Thacker Pass lithium mine are more supportive of the proposed mining project. This supports the YIMBY perspective (Hypothesis 1b). This may suggest that people living close to the Thacker Pass expect local-specific economic benefits such as new job opportunities and worry less about widely discussed costs that mine critics have pointed out.

**Table 1:** Main result.

	Coef.	SE	
Treatment			
Treatment 1	-0.101	0.133	
Treatment 2	0.238	0.131	*
Treatment 3	0.216	0.132	
Distance from TP	-0.001	0.001	**
Exposure	-0.059	0.079	
Environment	-0.959	0.106	***
Gender	-0.641	0.094	***
Age	0.012	0.003	***
Education	0.102	0.050	**
Income	-0.001	0.043	
Duration	-0.042	0.024	*
Party Identification			
Independent	-0.269	0.113	**
Republican	-0.449	0.118	***
Intercept	-17.960	5.520	***
*N*	1368		
Adjusted *R*^*2*^	0.113		

*Note*:

*: p < 0.1;

**: p < 0.05;

***: p < 0.01

Might geography play a role differently? It is plausible that individuals support Thacker Pass because they have not experienced the negative aspects of mining. We do not find support for this argument: the variable *Exposure* is not statistically significant (Hypothesis 2 is not supported). This might suggest that individuals are persuaded less by their prior experiences with existing mines in evaluating the benefits and costs in the future of a proposed mine.

When we turn to the effect of the treatment variable, only Treatment 2, the national security frame, increases support for the Thacker Pass mine. This suggests that respondents are sensitive to the national security dimension, a national-level public good, of the Thacker Pass project (Hypothesis 3b is supported).

However, neither climate benefits (global public good) nor local economic development benefits (local public good) change support for the Thacker Pass mine (Hypothesis 3a and 3c are not supported). Interestingly, while the coefficient of local economic development is positive, the coefficient of climate frame is negative (although neither achieves statistical significance). This raises issues about the political appeal of the climate issues which seemed to have had traction in Finland’s 2019 parliamentary elections [64], Switzerland’s 2019 federal elections [65], 2020 US Congressional elections [66], and Germany’s 2021 federal elections. In Brazil, protecting the Amazon, the critical component of climate mitigation which imposes local costs, is dominating the forthcoming Presidential elections. However, Rootes (2014) suggests that the carbon tax led to the Labor Party’s defeat in the 2013 Australian elections [67]. The Green Party fared poorly in Sweden’s 2018 Parliamentary Elections [68]. Clarke et al. (2011) suggest that the carbon tax issue hurt the Liberal Party in the 2018 Canadian federal elections [69]. Stokes (2016) finds that citizens living near wind energy projects punished the incumbent Liberal party in the 2011 Ontario provincial elections [70].

The lack of support for local economic development was unexpected. It challenges the received wisdom in American politics: “it’s the economy, stupid!” Indeed, many suggest that climate policies will have more traction if they are cast in terms of local jobs and employment (generating YIMBY dynamics), as opposed to the moral imperative to reduce greenhouse gas emissions. Hence, it is surprising that the local economic development did not increase support for the Thacker Pass project in relation to the reference category.

In examining respondent-specific attributes, we find that individuals with pro-environment views are *more* supportive of the Thacker Pass project in relation to pro-economic growth respondents. We speculate that these individuals probably see the Thacker Pass mine to be crucial for decarbonization and are willing to overlook its local environmental harm. Furthermore, while respondents with higher education and young people are more supportive of the lithium mine, income level does not affect the support levels. These results confirm the conventional notion that young people and individuals with higher education are more environmentally oriented [71]. The finding that women are less supportive of the mine may reflect that women tend to be more risk averse given the environmental harm caused by mining activities [72].

Arguably, given the large number of working mines in Nevada, the longer-term residents will recognize the economic contributions (or harms) of the mining sector and will be more (less) supportive of the mine. We find that the length of residence in Nevada decreases support for the mine, which suggests that exposure to mining sensitizes individuals to its negative local impacts. Lastly, partisanship effects, independents and Republicans are less supportive of the Thacker Pass mine in relation to democrats, which may reflect the fact that democrats have a greater environmental orientation than republicans.

## Additional analysis: Does geography moderate framing effects?

**Fig 4:**
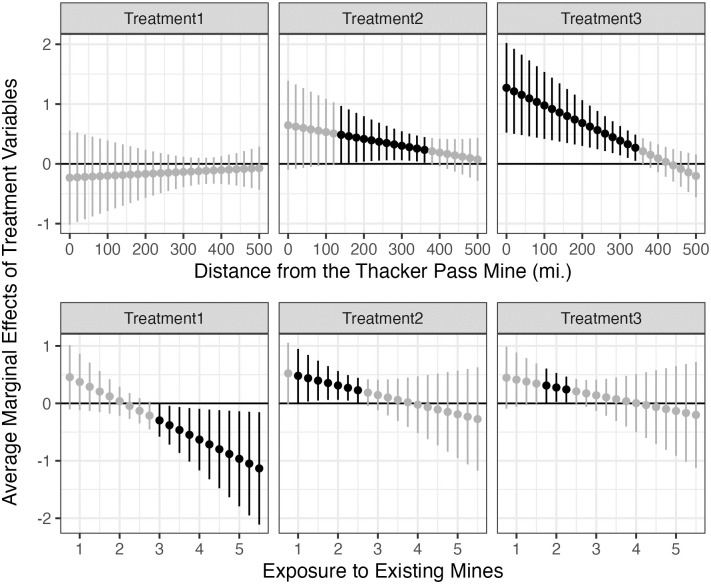
Conditional effect of *treatment* on *dist_TP* and *exposure*.

So far, we have explored the independent effect of treatment information on support for the Thacker Pass mine. Does distance to the Thacker Pass lithium mine or exposure to existing Nevada mines condition the effect of treatments? To explore this issue, we interacted *Treatment* with *Distance_TP* and *Treatment* with *Exposure* in separate models. The upper and lower panels of Fig 4 show the marginal effect of three treatment frames (with 90% confidence intervals), conditional on the distance to Thacker Pass and exposure to existing Nevada mines, respectively.

The upper panel shows that while the effect of the climate frame is not statistically significant regardless of where people live, the national security frame has a statistically significant effect among those who live within 175–350 miles from the Thacker Pass lithium mine (Fig 4). Even though local economic development had no independent effect on support for the Thacker Pass mine, it increases public support among respondents living close to the proposed mine (0–350 miles). This result may reflect that people living closer to the Thacker Pass lithium mine believe that they are more likely to receive economic benefits such as new jobs and an increase in tax revenues. Perhaps, the political dictum, “it’s the economy, stupid” does hold but only among people living close to the mine.

While exposure to existing mines had no independent effect in our main model, the lower panel of Fig 4 shows that it conditions the treatment effects of national security and economic development frames. When exposure to existing mines is low (1–1.5), the national security frame increases public support. Likewise, the local economic development frame increases public support when exposure to mines is relatively low (1.75–2.25). These results may suggest that individuals with limited first-hand knowledge about the benefits and costs of mining express higher support for the Thacker Pass project when they are told that it will help national security or support local economic development. Surprisingly, respondents with high exposure to mines (3–5.4) show lower support when treated with the climate frame. One possible explanation for this puzzling result is that exposure to climate information might trigger a perception of unfairness: local communities bear the costs of creating a global public benefit.

## Discussion

Decarbonization is being stymied by political conflicts rooted in the asymmetrical distribution of costs and benefits across actors, sectors, and communities. Some communities oppose renewable energy projects such as solar and wind farms and transmission lines because they bear costs such as landscape destruction and property value devaluation while most benefits are appropriated by others. In many U.S. counties, governments have enacted ordinances restricting the siting of renewable energy projects. This story is getting repeated in South Korea, India, Greece, Germany, Portugal, and the United Kingdom. There is local opposition to new transmission lines as well; consider opposition in New England states to new transmission lines to deliver hydropower from Quebec to New York.

This study explores public support for another type of decarbonization project: a lithium mine in Nevada. In examining this support, we considered where Nevada residents live in relation to the *proposed* lithium mine, and how proximity to any type of *existing* mine might shape their support. Further, we explore whether different framing of the benefits from the proposed mine might affect support levels. We find that support for the Thacker Pass mine is higher among those who live closer to the mine, lending support to the YIMBY argument. Proximity to existing mines, a proxy for first-hand knowledge of the costs and benefits of mining, does not change support levels. We also find that, unlike climate framing, national security framing increases public support for the Thacker Pass. And information about local economic benefits increases public support among only those who live close to the proposed mine.

Our paper has important implications for climate policy. While the “just transition” debate focuses on how decarbonization *policies* impose costs on fossil fuel communities, we explore how decarbonization *projects* impact local communities in ways that might activate both YIMBY and NIMBY dynamics [10, 73, 74]. These projects include infrastructure (such as solar/wind farms and new transmission lines) as well as supply chain (as in lithium, Nickel, and cobalt mines). Thus, we suggest that the scope of the just-transition debate could be extended to include both decarbonization policies and projects.

Second, our paper offers new ideas on how to navigate local opposition to decarbonization projects. We suggest that projects such as the proposed Thacker Pass mine might secure higher local support if they are justified on non-climate grounds such as national security (a national-level public good), and to some extent economic development (a local public good). Our findings support the literature that points out that in the U.S. decarbonization projects could get political support if they are not framed in terms of climate policy—which triggers political polarization.

Marshall and Burges (2022) find that Republican-controlled states enacted about one-third of state-level renewable energy legislation [75]. Texas, a conservative state, is the national leader in creating wind energy capacity. Indiana, another deep-red state, is constructing a 440 MW solar facility, Mammoth Solar, spread over 13,000 acres. At the opening ceremony, Indiana’s Republican Governor Eric Holcomb noted, "It’s an incredibly electrifying day for the state of Indiana as we celebrate Doral Renewables’ significant investment in the future of energy generation and the state of Indiana” [76]. Note the absence of the climate frame even for solar energy!

Recent disruptions in global supply chains, first due to COVID-19 and then due to the Ukraine invasion, have highlighted the need for the U.S. (western powers in general) to be self-sufficient for inputs critical for rapid decarbonization. While climate policy provokes partisan wars in the U.S., self-sufficiency in critical minerals, an important aspect of national security, has bipartisan support. China is a major player in the renewable energy supply chain. Russia is a key nickel supplier, an important component of rechargeable batteries. This suggests that policy makers can employ concerns about national security among the public to mobilize local support for opening new critical minerals mines and building renewable energy projects.

Our paper has several limitations. While we have accounted for respondents’ proximity to any of the 165 existing Nevada mines as a proxy for their first-hand exposure to mining, we have not examined whether respondents work in the mining industry or its supply chains such as trucking and construction. Future work should also explore whether the public reacts to the closure of existing mines differently from opening mines. Moreover, we have not controlled for proximity to closed mines. This is important because many mining companies have an abysmal record of leaving polluted wastelands once their mines are closed down.

Regarding the generalizability of our findings, support for new mines might differ in states where mining is less important. Further, Russia invaded Ukraine on February 24, 2022. Our survey was administered between February 24–March 1, 2022. Thus, it is possible that the salience of national security issues increased in this period which influenced our respondents.

Regarding local opposition to decarbonization projects, we recognize that the drivers of local opposition/support to new mines might differ from that of siting of solar or wind farms. The reason is that national security issues, especially, China’s control of the renewable energy supply chain, are probably more salient in mining issues. Thus, the national security-focused communication strategies that work well to enhance local support in mining projects, might be less effective for renewable energy siting. Thus, the generalizability of our findings outside mining to address local opposition to decarbonization should be carefully evaluated.

## Supporting information

S1 Appendix Online appendix for “Pursuing decarbonization along with national security: Assessing public support for the Thacker Pass lithium mine”. (DOCX)
