# Supplementary material for: Pursuing decarbonization along with national security: Assessing public support for the Thacker Pass lithium mine
Source: PLoS One. 2023 Jan 24;18(1):e0280720. doi: 10.1371/journal.pone.0280720 (PMC9873159; doi:10.1371/journal.pone.0280720)
Supplement: S1 Appendix — (DOCX) [file pone.0280720.s001.docx]

***Online Appendix***

***for***

***“Pursuing decarbonization along with national security: Assessing public support for the Thacker Pass lithium mine”***

**Table of Contents**

**Table A1.** Representativeness of our sample

**Table A2.** Result with attentive and inattentive respondents

**Table A3.** Result with an alternative specification of exposure

**Text A4.** Full text of survey questions and display instructions

**Table A1. Representativeness of our sample**

**By county**

| County | Respondents (%) | ACS (%) |
| --- | --- | --- |
| Carson City | 2.407 | 1.823 |
| Churchill | 1.170 | 0.812 |
| Clark | 72.661 | 73.553 |
| Douglas | 1.243 | 1.600 |
| Elko | 1.023 | 1.734 |
| Esmeralda | 0.073 | 0.034 |
| Eureka | 0.000 | 0.061 |
| Humboldt | 0.512 | 0.556 |
| Lander | 0.146 | 0.184 |
| Lincoln | 0.877 | 0.171 |
| Lyon | 2.047 | 1.837 |
| Mineral | 0.146 | 0.148 |
| Nye | 2.210 | 1.502 |
| Pershing | 0.146 | 0.218 |
| Storey | 0.000 | 0.135 |
| Washoe | 15.351 | 15.318 |
| White Pine | 0.439 | 0.316 |

**By gender**

| Gender | Respondents (%) | ACS (%) |
| --- | --- | --- |
| Male | 42.763 | 50.167 |
| Female | 57.237 | 49.833 |

**By gender x county**

| County | Gender | Respondents (%) | ACS (%) |
| --- | --- | --- | --- |
| Carson City | Male | 0.365 | 0.939 |
|  | Female | 1.681 | 0.884 |
| Churchill | Male | 0.439 | 0.417 |
|  | Female | 0.731 | 0.395 |
| Clark | Male | 32.602 | 36.686 |
|  | Female | 40.058 | 36.868 |
| Douglas | Male | 0.512 | 0.807 |
|  | Female | 0.731 | 0.793 |
| Elko | Male | 0.365 | 0.905 |
|  | Female | 0.658 | 0.828 |
| Esmeralda | Male | 0.073 | 0.016 |
|  | Female | 0.000 | 0.018 |
| Eureka | Male | 0.000 | 0.031 |
|  | Female | 0.000 | 0.030 |
| Humboldt | Male | 0.146 | 0.289 |
|  | Female | 0.365 | 0.266 |
| Lander | Male | 0.073 | 0.100 |
|  | Female | 0.073 | 0.084 |
| Lincoln | Male | 0.292 | 0.096 |
|  | Female | 0.585 | 0.075 |
| Lyon | Male | 0.731 | 0.924 |
|  | Female | 1.316 | 0.913 |
| Mineral | Male | 0.000 | 0.070 |
|  | Female | 0.146 | 0.078 |
| Nye | Male | 0.804 | 0.769 |
|  | Female | 1.316 | 0.733 |
| Pershing | Male | 0.073 | 0.139 |
|  | Female | 0.073 | 0.078 |
| Storey | Male | 0.000 | 0.071 |
|  | Female | 0.000 | 0.064 |
| Washoe | Male | 6.140 | 7.719 |
|  | Female | 9.211 | 7.599 |
| White Pine | Male | 0.146 | 0.189 |
|  | Female | 0.292 | 0.127 |

**By age x county**

| County | Age | Respondents (%) | ACS (%) |
| --- | --- | --- | --- |
| Carson City | 18 to 39 | 0.804 | 0.628 |
|  | 40 to 59 | 0.585 | 0.597 |
|  | 60 to | 0.658 | 0.652 |
| Churchill | 18 to 39 | 0.365 | 0.282 |
|  | 40 to 59 | 0.585 | 0.270 |
|  | 60 to | 0.219 | 0.260 |
| Clark | 18 to 39 | 26.170 | 28.805 |
|  | 40 to 59 | 23.099 | 24.926 |
|  | 60 to | 23.392 | 19.353 |
| Douglas | 18 to 39 | 0.219 | 0.420 |
|  | 40 to 59 | 0.512 | 0.517 |
|  | 60 to | 0.512 | 0.791 |
| Elko | 18 to 39 | 0.365 | 0.682 |
|  | 40 to 59 | 0.512 | 0.579 |
|  | 60 to | 0.146 | 0.379 |
| Esmeralda | 18 to 39 | 0.073 | 0.009 |
|  | 40 to 59 | 0.000 | 0.012 |
|  | 60 to | 0.000 | 0.017 |
| Eureka | 18 to 39 | 0.000 | 0.015 |
|  | 40 to 59 | 0.000 | 0.019 |
|  | 60 to | 0.000 | 0.023 |
| Humboldt | 18 to 39 | 0.219 | 0.188 |
|  | 40 to 59 | 0.146 | 0.174 |
|  | 60 to | 0.146 | 0.166 |
| Lander | 18 to 39 | 0.073 | 0.062 |
|  | 40 to 59 | 0.073 | 0.068 |
|  | 60 to | 0.000 | 0.044 |
| Lincoln | 18 to 39 | 0.512 | 0.062 |
|  | 40 to 59 | 0.146 | 0.054 |
|  | 60 to | 0.219 | 0.063 |
| Lyon | 18 to 39 | 0.731 | 0.576 |
|  | 40 to 59 | 0.658 | 0.616 |
|  | 60 to | 0.658 | 0.680 |
| Mineral | 18 to 39 | 0.000 | 0.047 |
|  | 40 to 59 | 0.073 | 0.041 |
|  | 60 to | 0.073 | 0.073 |
| Nye | 18 to 39 | 0.219 | 0.373 |
|  | 40 to 59 | 0.658 | 0.479 |
|  | 60 to | 1.243 | 0.762 |
| Pershing | 18 to 39 | 0.146 | 0.085 |
|  | 40 to 59 | 0.000 | 0.083 |
|  | 60 to | 0.000 | 0.066 |
| Storey | 18 to 39 | 0.000 | 0.035 |
|  | 40 to 59 | 0.000 | 0.043 |
|  | 60 to | 0.000 | 0.068 |
| Washoe | 18 to 39 | 4.240 | 5.980 |
|  | 40 to 59 | 5.117 | 5.022 |
|  | 60 to | 5.994 | 4.524 |
| White Pine | 18 to 39 | 0.073 | 0.122 |
|  | 40 to 59 | 0.219 | 0.108 |
|  | 60 to | 0.146 | 0.100 |

**Table A2. Result with attentive and inattentive respondents**

|  | Coef. | SE | |
| --- | --- | --- | --- |
| Treatment |  |  |  |
| Treatment 1 | -0.107 | 0.128 |  |
| Treatment 2 | 0.207 | 0.125 | ^*^ |
| Treatment 3 | 0.151 | 0.127 |  |
| Distance from TP | -0.001 | 0.001 | ^**^ |
| Exposure | -0.063 | 0.076 |  |
| Environment | -0.880 | 0.101 | ^***^ |
| Gender | -0.628 | 0.091 | ^***^ |
| Age | 0.012 | 0.003 | ^***^ |
| Education | 0.106 | 0.047 | ^**^ |
| Income | 0.008 | 0.041 |  |
| Duration | -0.048 | 0.023 | ^**^ |
| Party Identification |  |  |  |
| Independent | -0.312 | 0.110 | ^***^ |
| Republican | -0.425 | 0.113 | ^***^ |
| Intercept | -18.771 | 5.274 | ^***^ |
| *N* | 1465 | | |
| Adjusted *R^2^* | 0.103 | | |

*Note*: *: p < 0.1; **: p < 0.05; ***: p < 0.01

**Table A3. Result with an alternative specification of exposure**

|  | Coef. | SE | |
| --- | --- | --- | --- |
| Treatment |  |  |  |
| Treatment 1 | -0.102 | 0.133 |  |
| Treatment 2 | 0.237 | 0.131 | ^*^ |
| Treatment 3 | 0.217 | 0.132 |  |
| Distance from TP | -0.001 | 0.001 |  |
| Exposure (County) | -0.003 | 0.013 |  |
| Environment | -0.958 | 0.106 | ^***^ |
| Gender | -0.640 | 0.094 | ^***^ |
| Age | 0.012 | 0.003 | ^***^ |
| Education | 0.100 | 0.050 | ^**^ |
| Income | -0.002 | 0.043 |  |
| Duration | -0.042 | 0.024 | ^*^ |
| Party Identification |  |  |  |
| Independent | -0.263 | 0.113 | ^**^ |
| Republican | -0.448 | 0.118 | ^***^ |
| Intercept | -17.969 | 5.522 | ^***^ |
| *N* | 1368 | | |
| Adjusted *R^2^* | 0.112 | | |

*Note*: *: p < 0.1; **: p < 0.05; ***: p < 0.01

**Text A4. Full text of survey questions and display instructions**

**Welcome page**

Welcome to this survey!

This survey focuses on **the mining policy in Nevada**. It should take around 8 minutes to complete. It will be used solely for academic research and is not funded by any commercial or governmental organization.

The survey is anonymous. The information you provide will not be stored or used in any way that could reveal your personal identity. There are no known risks posed by participating in this survey. The survey has been reviewed by University of Washington's Human Subject Division (STUDY00014935). Your participation is voluntary, and you may discontinue participating in the survey at any time.

**This survey will produce meaningful results only if you read questions carefully and express your true opinion.**

If you have any questions about this study, please email Prof. Nives Dolsak, University of Washington, [nives@uw.edu](mailto:nives@uw.edu). By completing this survey, you are consenting to participate in this study. We are grateful for your participation.

[Page break]

Please read the following text **very carefully** and then answer the question.

**Information frames**

**Control**

The automobile industry is in the midst of a major transition. Governments and auto companies want to phase out the internal combustion engine in favor of electric vehicles (EVs). This means that instead of driving up to a gas station, drivers will plug in their vehicles to recharge at home, at work, while shopping, or in public parking. The key motivation for this transformation is **climate change**. If cars can run on electricity and electricity can be generated by zero-emission sources such as solar, wind or nuclear, greenhouse gas emissions will be substantially reduced. However, **EVs require powerful rechargeable batteries, which need lithium.** This is why there is an increased demand for lithium worldwide. By some estimates, lithium’s global demand will grow at least 13-fold by 2040.

**There is a proposal for a lithium mine in Thacker Pass, Humboldt County, Nevada.** This has generated some controversy. Native American groups have opposed it because it will disturb their sacred burial grounds. Environmental groups are worried that **lithium processing facilities cause harm to endangered trout, sensitive sage-grouse and antelope, and an increase in local air and water pollution**. Finally, some note that lithium mining needs a substantial amount of **water, which is short in supply in Nevada**. (207 words)

**Frame 1: Climate Policy**

The automobile industry is in the midst of a major transition. Governments and auto companies want to phase out the internal combustion engine in favor of electric vehicles (EVs). The key motivation for this transformation is **climate change**. If cars can run on electricity, greenhouse gas emissions will be substantially reduced. However, **EVs require powerful rechargeable batteries, which need lithium.** By some estimates, lithium’s global demand will grow at least 13-fold by 2040.

**There is a proposal for a lithium mine in Thacker Pass, Humboldt County, Nevada.** Native American groups have opposed it because it will disturb their sacred burial grounds. Environmental groups are worried that **lithium processing facilities cause harm to endangered trout, sensitive sage-grouse and antelope, and an increase in local air and water pollution**. Finally, some note that lithium mining needs a substantial amount of **water, which is short in supply in Nevada**.

The United States, **the largest contributor of accumulated greenhouse gas emissions**, has pledged to substantially reduce these emissions. Transportation activities, mostly associated with passenger cars, account for about 30% of U.S. emissions. Moreover, transportation emissions continue to grow. Since the United States has sizeable lithium deposits, it should facilitate lithium mining **to support reductions in greenhouse gas emissions**. (206 words)

**Frame 2: China Competition**

The automobile industry is in the midst of a major transition. Governments and auto companies want to phase out the internal combustion engine in favor of electric vehicles (EVs). The key motivation for this transformation is **climate change**. If cars can run on electricity, greenhouse gas emissions will be substantially reduced. However, **EVs require powerful rechargeable batteries, which need lithium.** By some estimates, lithium’s global demand will grow at least 13-fold by 2040.

**There is a proposal for a lithium mine in Thacker Pass, Humboldt County, Nevada.** Native American groups have opposed it because it will disturb their sacred burial grounds. Environmental groups are worried that **lithium processing facilities cause harm to endangered trout, sensitive sage-grouse and antelope, and an increase in local air and water pollution**. Finally, some note that lithium mining needs a substantial amount of **water, which is short in supply in Nevada**.

**The United States and China are competing in new technologies**. China is the leader in the EV sector, including lithium batteries. To remain globally competitive in the automobile sector, the United States needs new lithium mines**.** Moreover, the United States and China are **military competitors**. Thus, for its national security reasons, the United States needs new lithium mines **to reduce reliance on China**. (211 words)

**Frame 3: Local Economic Development**

The automobile industry is in the midst of a major transition. Governments and auto companies want to phase out the internal combustion engine in favor of electric vehicles (EVs). The key motivation for this transformation is **climate change**. If cars can run on electricity, greenhouse gas emissions will be substantially reduced. However, **EVs require powerful rechargeable batteries, which need lithium.** By some estimates, lithium’s global demand will grow at least 13-fold by 2040.

**There is a proposal for a lithium mine in Thacker Pass, Humboldt County, Nevada.** Native American groups have opposed it because it will disturb their sacred burial grounds. Environmental groups are worried that **lithium processing facilities cause harm to endangered trout, sensitive sage-grouse and antelope, and an increase in local air and water pollution**. Finally, some note that lithium mining needs a substantial amount of **water, which is short in supply in Nevada**.

Lithium mining will attract a **large volume of investments and create well-paying local jobs** both in the construction phase and in mining and processing facilities. It will help the local economy, such as the retail sector, by **boosting economic activities**. The lithium facility will also increase **tax revenue for local governments** which will allow them to fund schools, roads, and public services. (208 words)

[Page break]

**Attention checks**

We want to make sure that you are paying attention to the information provided in this survey. Please select the topic you read about on the previous page.

A1:

- Rechargeable batteries for EVs require copper.
- Rechargeable batteries for EVs require potassium.
- Rechargeable batteries for EVs require lithium.

A2:

- There is a proposal for a new mining project in Humboldt County.
- There is a proposal for a new mining project in Elko County.
- There is a proposal for a new mining project in Lander County.

[Page break]

**Main question**

Please indicate your support for the Thacker Pass Lithium mine, by moving the slider bar.

Support Level

Strongly oppose Neither support nor oppose Strongly support

1 2 3 4 5 6 7

[Page break]

**Mechanism questions**

Q. Criteria

You have just indicated your level of support for the Thacker Pass mine. How ***important*** were the following factors in shaping your response?

- Lithium is critical for reducing carbon emissions.
- The US must take responsibility for reducing its carbon emissions.
- Mining causes local environmental pollution, water scarcity, and harms wildlife.
- China is a military and economic competitor to the US.
- Mining is critical for the economic prosperity of Nevada.

1. Not at all important
2. Slightly important
3. Fairly important
4. Very important

[Page break]

**Attitude questions**

Q. Environmental Attitude

Here are two statements people sometimes make when discussing the relationship between environmental protection and economic growth. Which of them is closer to your viewpoint?

A. Environmental protection should have priority over economic growth.

B. Economic growth should be given priority over environmental protection.

(1) Statement A

(2) Statement B

[Page break]

**Perceived Proximity**

Q. Existing mining sites -location

To the best of your knowledge, do you know whether any mines (gold, silver, sand, gravel, rock, clay, limestone, sodium, potassium, lithium, gemstone, and oil etc.) are located in your county?

1. Yes
2. No
3. Don’t know

Q. Existing mining sites -effect

For respondents who answer yes above:

Overall, in your assessment, is mining beneficial for your community?

1. Yes
2. No
3. Don’t know/Can’t say.

[Page break]

**Demographic questions**

Q. Age

When were you born? (Please enter your year of birth.)

(if respondents choose more than 2003, show “You must be 18 or older.”)

Q. Gender

What is your gender? [Provide pull-down menu]

(1) Male

(2) Female

(3) Non-binary

(4) Prefer not to answer

[Page break]

Q. Race/ethnicity

1. Please specify your race/ethnicity. You can select more than one.
2. African-American
3. Caucasian
4. Latino or Hispanic
5. Asian
6. Native American
7. Native Hawaiian or Pacific Islander
8. Other/Unknown
9. Prefer not to answer.

Q. Religion

What is your religious preference?

1. Protestant
2. Catholic
3. Orthodox
4. Other Christians
5. Jewish
6. Muslim
7. Hindu
8. Buddhist
9. Other faiths
10. Not religious
11. Don’t know/prefer not to say

[Page break]

Q. Location

Which ZIP code do you currently live in?

Q. Duration of residence

How long have you lived in Nevada? [Provide pull-down menu]

1. Less than 1 year
2. 1-5 years
3. 5-10 years
4. 10-15 years
5. 15-20 years
6. 20-25 years
7. More than 25 years

[Page break]

Q. Education

What best describes the highest level of education you completed?

1. Middle School or below
2. High school incomplete
3. High school
4. Some college, no degree
5. Four-year college
6. Graduate school

Q. Employment status

Are you currently employed?

(1) Yes (this includes self-employment)

(2) No, but I am actively looking for a job

(3) No, I am a student

(4) No, I am retired

(5) No, I am taking care of the household

(6) Other

[Page break]

Q. Household income

What is your annual ***household*** income before taxes? Like the rest of the survey, this question is completely confidential and will be used only to classify the survey responses.

[Provide pull-down menu]

(1) Less than $40,000

(2) $40,000 - $69,999

(3) $70,000 - $104,999

(4) $105,000 - $159,999

(5) $160,000 or higher

(-99) Prefer not to answer

Q. Political affiliation

In the U.S., many people lean towards a particular party, although they may occasionally vote for a candidate from a different party. How about you; do you in general lean towards a particular party? If so, which one?

[Randomize order of items]

(1) Republican

(2) Democrat

(3) Independent

(-99) Prefer not to answer

[Page break]

We appreciate your participation.
